# The influence of maternal androgen excess on the male reproductive axis

**DOI:** 10.1038/s41598-019-55436-9

**Published:** 2019-12-11

**Authors:** Sarah Holland, Melanie Prescott, Michael Pankhurst, Rebecca E. Campbell

**Affiliations:** 1 Centre for Neuroendocrinology and Department of Physiology, School of Biomedical Sciences, University of Otago, Dunedin, 9054 New Zealand; 2 Department of Anatomy, School of Biomedical Sciences, University of Otago, Dunedin, 9054 New Zealand

**Keywords:** Neural circuits, Polycystic ovary syndrome, Neuroendocrine diseases

## Abstract

Prenatal androgen excess is suspected to contribute to the development of polycystic ovary syndrome (PCOS) in women. Evidence from preclinical female animal models links maternal androgen excess with the development of PCOS-like features and associated alterations in the neuronal network regulating the reproductive axis. There is some evidence suggesting that maternal androgen excess leads to similar reproductive axis disruptions in men, despite the critical role that androgens play in normal sexual differentiation. Here, the specific impact of maternal androgen excess on the male hypothalamic-pituitary-gonadal axis was investigated using a prenatal androgenization protocol in mice shown to model PCOS-like features in females. Reproductive phenotyping of prenatally androgenised male (PNAM) mice revealed no discernible impact of maternal androgen excess at any level of the reproductive axis. Luteinising hormone pulse characteristics, daily sperm production, plasma testosterone and anti-Müllerian hormone levels were not different in the male offspring of dams administered dihydrotestosterone (DHT) during late gestation compared to controls. Androgen receptor expression was quantified through the hypothalamus and identified as unchanged. Confocal imaging of gonadotropin-releasing hormone (GnRH) neurons revealed that in contrast with prenatally androgenised female mice, PNAM mice exhibited no differences in the density of putative GABAergic innervation compared to controls. These data indicate that a maternal androgen environment capable of inducing reproductive dysfunction in female offspring has no evident impact on the reproductive axis of male littermates in adulthood.

## Introduction

The prenatal environment of the developing foetus is critical in shaping future adult health and fertility. This theory of a foetal origin of adult disease, known as the Barker Hypothesis^1^, asserts that adverse influences during intrauterine development can predispose an individual to developing disease in adulthood, such as cardiovascular^2^, metabolic^3^ and reproductive^4,5^ pathologies. The developing reproductive system is acutely sensitive to the nutritional and hormonal environment *in utero*, and disruptions within this environment can elicit reproductive dysfunction in offspring^5–7^. For example, the exposure of female foetuses to elevated maternal androgen levels is suspected to contribute to the development of some forms of polycystic ovary syndrome (PCOS)^8,9^, a prevalent female neuroendocrine disorder^8,10^. Notably, there is also some evidence linking an elevated maternal androgen environment to similar disruptions in the reproductive axis of males^11,12^. However, given the critical role that foetal testosterone plays in normal sexual differentiation of males, it is not clear how maternal androgen excess impacts the function of the adult male reproductive axis.

The reproductive deficits that develop in female offspring exposed to androgen excess are postulated to result from modifications to the neuronal network that regulates fertility. Gonadotropin-releasing hormone (GnRH) neurons, distributed throughout the basal forebrain^13^, project caudally to secrete the GnRH peptide into the median eminence. This release of GnRH in turn elicits the secretion of luteinising hormone (LH) and follicle-stimulating hormone (FSH) from the anterior pituitary gland^14^. In both males and females, gonadal steroid hormones provide critical feedback information to GnRH neurons via an afferent, steroid hormone-sensitive neuronal network^15^. PCOS patients frequently exhibit impaired steroid hormone negative feedback and a hyperactive GnRH/LH secretion profile^16–18^, suggesting pathological wiring within the GnRH neuronal network.

Preclinical models of PCOS have highlighted the critical role of the brain in androgen-mediated disruption of female fertility^19^. Female mice prenatally exposed to the non-aromatisable androgen di-hydrotestesterone (prenatally androgenised (PNA) mouse model) manifest the cardinal reproductive deficits of PCOS seen in women, and associated impairments in steroid hormone feedback^20–22^. Modifications in the GnRH neuronal network, including significantly enhanced GABAergic synaptic input^23,24^ and signalling^20^ to GnRH neurons, have been correlated with PCOS-like traits observed in PNA females. While elevated androgen signalling during development induces reprogramming of brain circuitry underlying reproductive function in females, the impact of a similar in utero environment on the adult male hypothalamic-pituitary-gonadal axis (HPG) axis remains unclear.

The existence of a male PCOS-like equivalent is suggested by clinical studies demonstrating that male relatives of women with PCOS present an array of similar reproductive and metabolic abnormalities to those seen in their female PCOS counterparts^11^. Male PCOS relatives are reported to exhibit elevated levels of hormones related to gonadal function, including LH, FSH and AMH, indicative of increased Sertoli cell number or function^25,26^. This reported increase suggests altered testicular function and altered neuroendocrine regulation of gonadotropin secretion^26^. While both genes and environment likely contribute to the familial nature of these features, preclinical studies in the male monkeys^27^ and rams^28–30^ indicate a detrimental effect of maternal androgen excess on metabolic and reproductive function respectively. Rams exposed to excess prenatal androgens have reduced sperm count, motility and concentration^28–30^ and increased testicular mRNA expression of AMH and SOX9^31^ in adulthood. These rams also exhibit a disrupted neuroendocrine axis, including significantly elevated FSH^32^, LH and testosterone^33^, and a significantly reduced testosterone to LH ratio in response to GnRH analog administration^32^. These findings suggest that in addition to the normal sexual differentiation and masculinisation that takes place downstream from endogenous foetal androgen production^34^, maternal androgen excess can have pathological consequences on the developing male HPG axis.

The purpose of the present study was to determine whether exposure of foetal male mice to maternal androgen excess programmes similar changes in the adult male reproductive axis to those seen in females. Testicular function, gonadal and pituitary hormone secretion and putative GABAergic inputs to GnRH neurons were assessed in adult transgenic GnRH-GFP male offspring of dams delivered DHT in late gestation.

## Results

### Testicular function is unaltered in adult males exposed prenatally to maternal androgen excess

**Figure 1:**
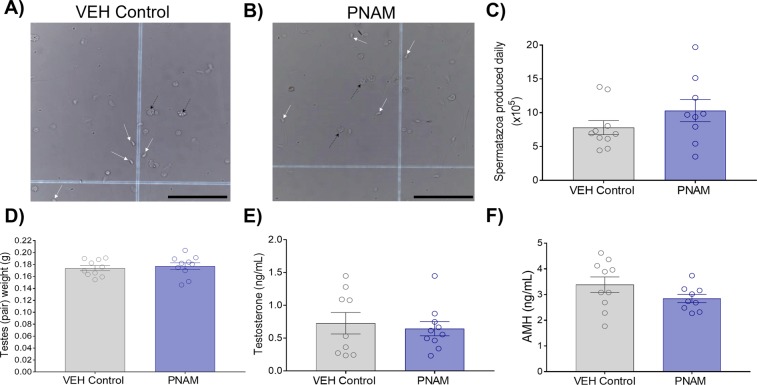
Daily spermatozoa production (DSP), testes weight, plasma testosterone and AMH levels are similar between VEH control and PNAM mice. Representative brightfield images (20× magnification, scale bars = 100 μm) of spermatozoa heads (indicated by white arrows) counted on haemocytometer grids from VEH control (**A**) and PNAM (**B**) mice. No differences were observed between PNAM (n = 9–10) and VEH control (n = 9–10) in DSP (**C**, Student’s t-test), testes weight (**D**, Student’s t-test), plasma testosterone levels (**E**, Wilcoxon rank sum test) or plasma AMH levels (**F**, Student’s t-test). Black arrows indicate cellular debris.

Daily sperm production (DSP) was similar between adult VEH control and PNAM mice (Fig. 1A–C; *p* = 0.20). Combined testis weight was also similar between VEH control (n = 10) and PNAM (n = 10) mice (Fig. 1D, *p* = 0.64), as was adult body weight (data not shown, Student’s t test, *p* = 0.89). Plasma testosterone levels were not different between PNAM (n = 10) and VEH control (n = 9) mice (Fig. 1E; *p* = 1.00). Likewise, plasma AMH levels were similar between PNAM (n = 9) and VEH control (n = 10) mice (Fig. 1F; *p* = 0.14).

### Maternal androgen excess does not affect AMH  or AMH receptor mRNA expression in the testes

**Figure 2:**
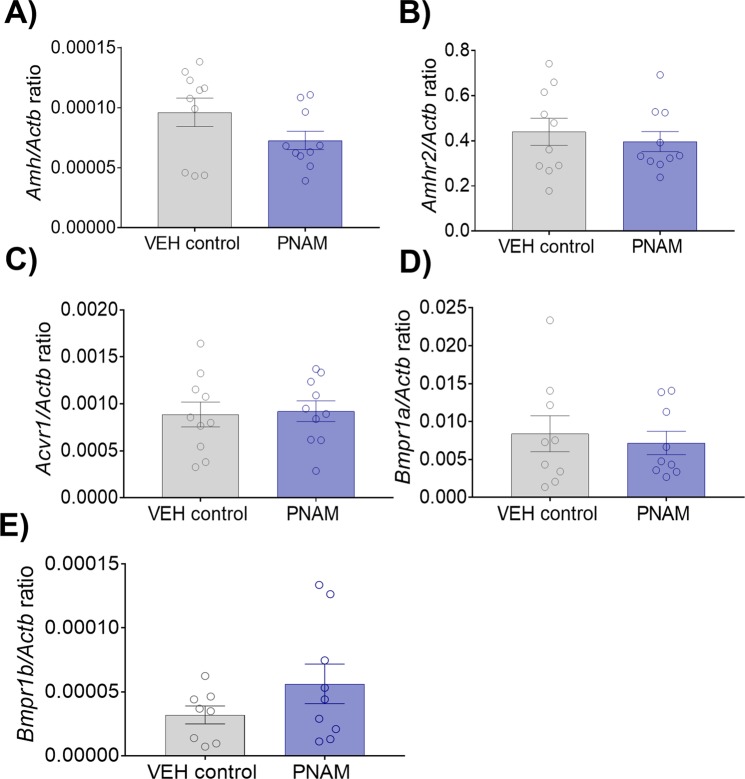
*Amh*, *Amhr2* and *Amh* type 1 receptor gene expression in testis were not altered in PNAM mice. Bar graphs that illustrate that the mean ± SEM relative mRNA expression/β-actin ratio for *Amh* (**A**, Student’s t-test), *Amhr2* (**B**, Student’s t-test), *Acvr1* (**C**, Student’s t-test), *Bmpr1a* (**D**, Wilcoxon rank sum test) and *Bmpr1b* (**E**, Wilcoxon rank sum test) was not different between VEH control (n = 8 to 10) and PNAM (n = 9 to 10).

Given previous reports that rams exposed to elevated prenatal androgens exhibit increased testicular mRNA expression of AMH^31^, we investigated whether PNAM mice also manifest an alteration in testicular mRNA expression of AMH and its receptor variants. qPCR was performed to detect mRNA expression of testicular *Amh*, AMH type 2 receptor (*Amhr2*) and variants of the AMH type 1 receptor (*Acvr1*, *Bmpr1a* and *Bmpr1b*). The relative mRNA expression for testicular *Amh* was similar between VEH control (n = 10) and PNAM (n = 10) mice (Fig. 2A; *p* = 0.11). Additionally, the relative mRNA expression was similar between VEH control (n = 10) and PNAM (n = 10) for testicular *Amhr2* (Fig. 2B; *p* = 0.57). The relative mRNA expression of the *Amh* type 1 receptor genes was also similar between VEH control and PNAM mice; *Acvr1* (Fig. 2C; VEH control, n = 10; PNAM, n = 10; *p* = 0.84), *Bmpr1a* (Fig. 2D; VEH control, n = 9; PNAM, n = 9; *p* = 0.67), *Bmpr1b* (Fig. 2E; VEH control, n = 8; PNAM, n = 9; *p* = 0.18).

### Male luteinising hormone (LH) pulse characteristics are not altered by maternal prenatal androgen excess

**Figure 3:**
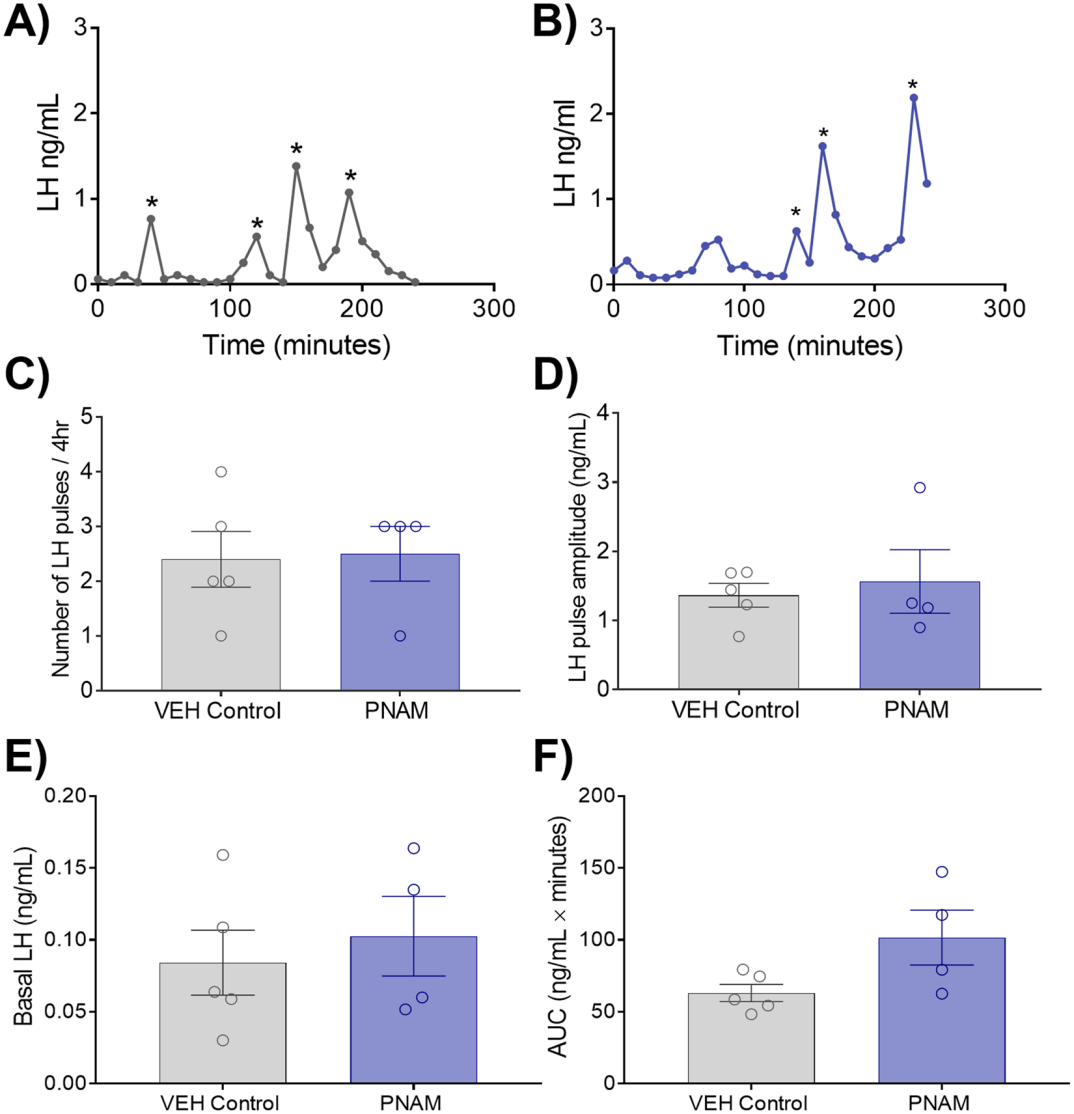
Pulsatile LH secretion is not different in prenatally androgenised male mice. Representative LH pulse traces from VEH control (**A**) and PNAM mice (**B**). Bar graphs illustrate mean ± SEM LH pulse frequency (**C**, Student’s t-test), LH pulse amplitude (**D**, Student’s t-test), basal LH levels (**E**, Student’s t-test) and area under the curve (AUC) (**F**, Student’s t-test) in VEH control (n = 5) and PNAM (n = 4) mice.

Serial tail-tip blood samples were collected over four hours to measure pulsatile LH secretion, as previously performed by our group^23^, in VEH control (n = 10) and PNAM (n = 10) mice. LH pulses, confirmed by a 255% increase in LH^35^, were only observed in 5 VEH and 4 PNAM mice, from whom LH pulse characteristics were compared. LH pulse-frequency, as determined by the number of LH pulses in 4 hours^35^ (Fig. 3A,B), was not different between VEH control and PNAM mice (Fig. 3C; *p* = 0.89). Additionally, LH pulse amplitude was similar between VEH control and PNAM mice (Fig. 3D; *p* = 0.67). Likewise, mean basal LH levels (Fig. 3e; *p* = 0.62) and area under the curve (AUC) (Fig. 3F; *p* = 0.07) was also not different between VEH control and PNAM mice.

### Adult hypothalamic androgen receptor expression is not affected by maternal androgen excess

**Figure 4:**
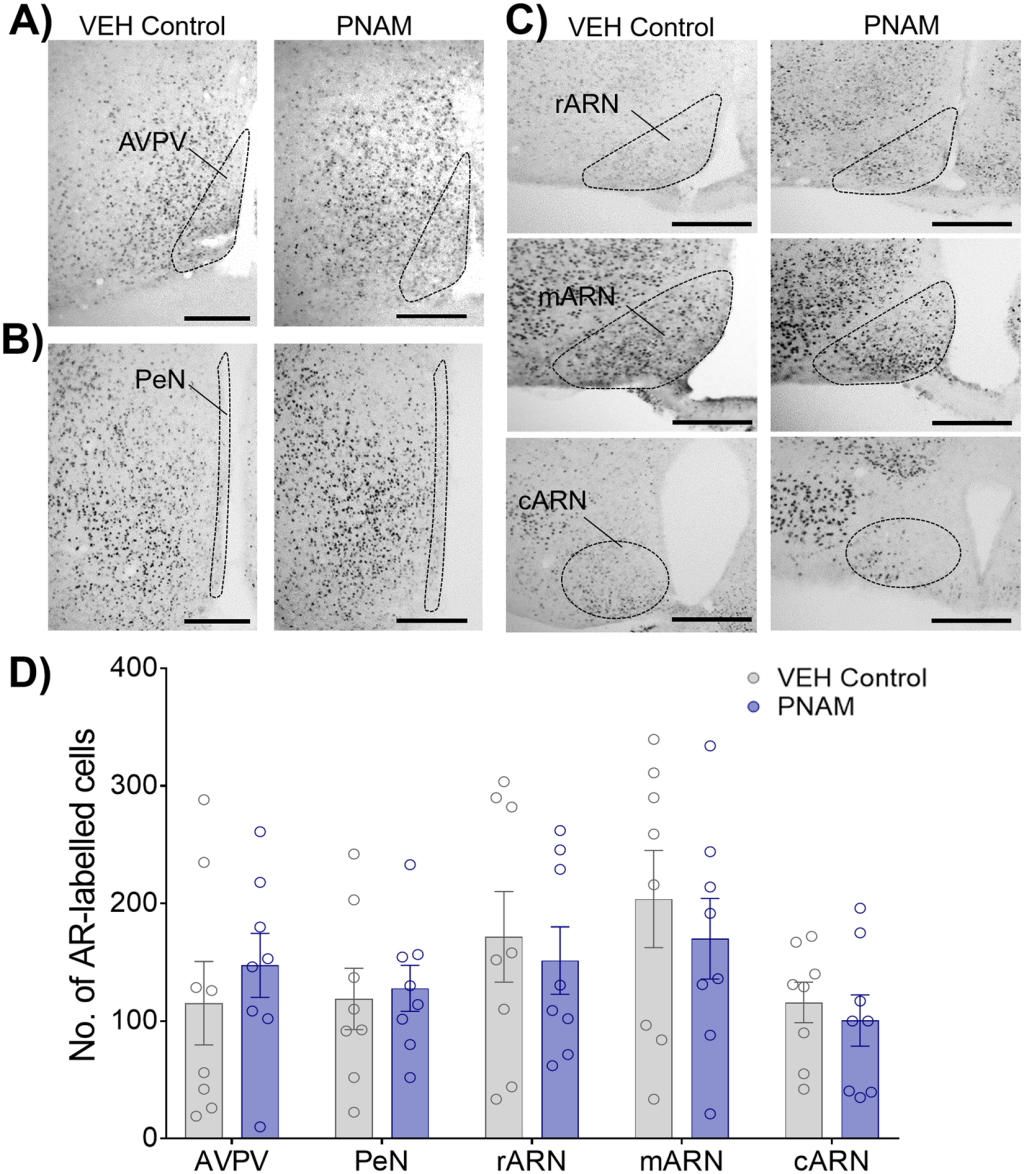
AR expression within the AVPV, PeN and regions of the ARN was not affected by prenatal androgen exposure. AR immunoreactivity in representative unilateral sections containing the AVPV (outlined, **A**), PeN (outlined, **B**), and rARN, mARN and cARN (outlined, **C**) in VEH control (n = 8) and PNAM (n = 8) mice. (Scale bar = 200 μm). The mean ± SEM number of AR-labeled cells was not significantly different in PNAM mice compared with controls (**D**, Student’s t-tests).

Maternal androgen excess impacts adult steroid hormone receptor expression in female offspring^23^. The expression of androgen receptors (AR) was assessed in PNAM mice in hypothalamic regions populated by GnRH neuronal afferent inputs^36^, including the anteroventral periventricular nucleus (AVPV), periventricular nucleus (PeN), rostral arcuate nucleus (rARN), middle arcuate nucleus (mARN) and caudal arcuate nucleus (cARN). The number of AR-positive cells was not different between VEH control (n = 8) and PNAM (n = 8) mice in any of the areas investigated. This included the AVPV (Fig. 4A,D; *p* = 0.48), the PeN (Fig. 4B,D; *p* = 0.79), and all regions of the arcuate nucleus (ARN) examined; the rARN (Fig. 4C,D; *p* = 0.68), the mARN (Fig. 4C,D; *p* = 0.54) and the cARN (Fig. 4C,D; *p* = 0.59).

### Maternal androgen excess does not affect putative GABAergic input to GnRH neurons in male offspring

**Figure 5:**
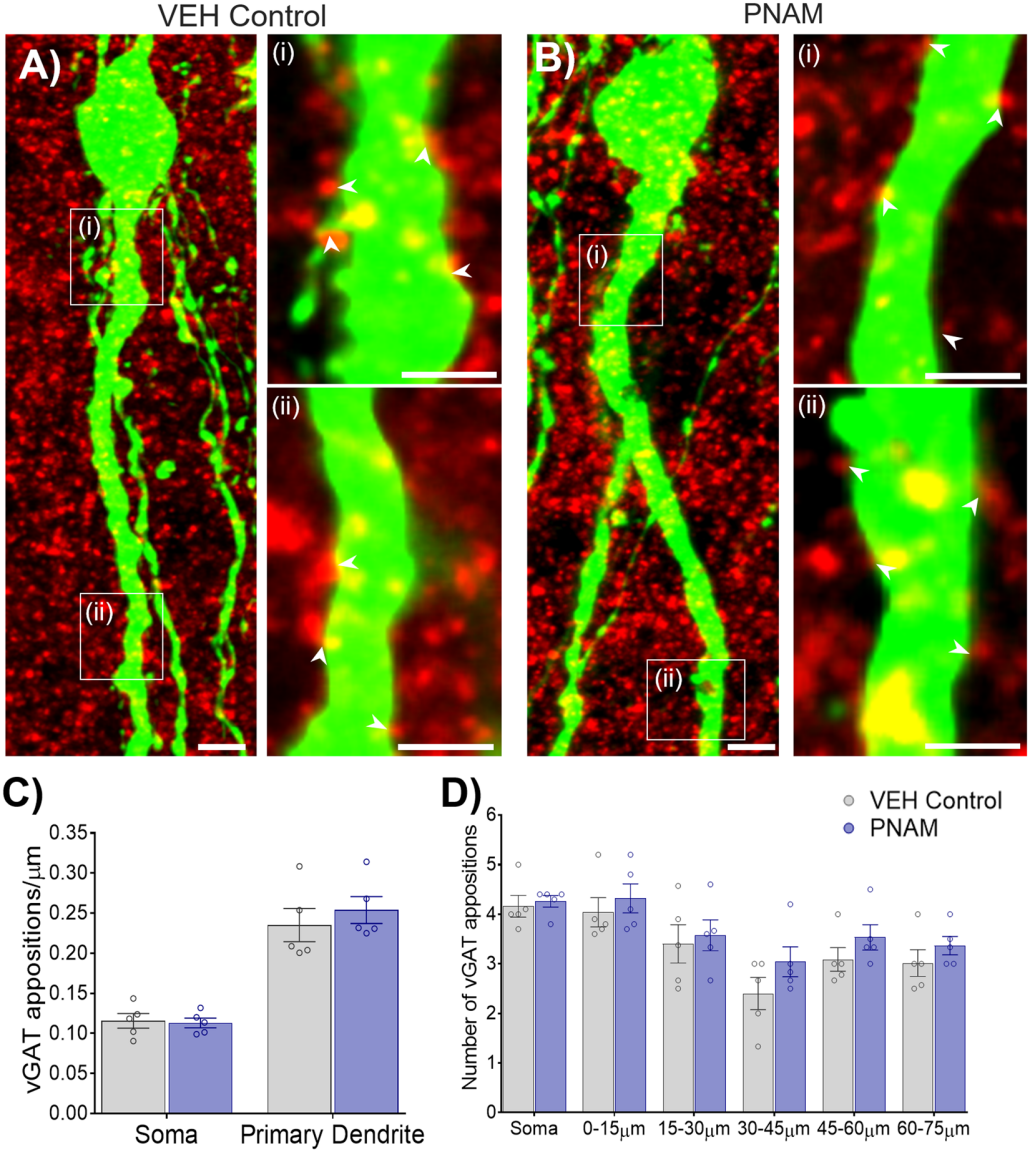
Putative GABAergic input to GnRH neurons was not affected by prenatal androgen exposure (PNA) in males. Projected Z-stacked confocal images of GFP-immunoreactive (-ir) GnRH neurons (green) surrounded by vGAT-ir puncta (red) from VEH control (**A**) and PNAM (**B**) mice (13 µm optical thickness). Dendritic segments of the primary dendrite from corresponding white boxes (i, ii) are shown in magnified confocal images (1.8 µm optical thickness). White arrowheads indicate vGAT-ir puncta considered to be apposing the GnRH neuron. Scale bars = 5 µm. Bar graphs (mean ± SEM) which depict the mean density of vGAT appositions to the GnRH cell soma and primary dendrite was not different between PNAM (n = 5) and VEH control (n = 5) mice (**C**, Student’s t-tests). Additionally, the raw number of counted vGAT appositions to the soma and along 15 µm segments of the primary dendrite was not different between VEH control and PNAM mice.

In order to determine whether maternal androgen excess enhances GABAergic inputs to GnRH neurons in male mice as seen in PNA females^23^, the number of closely apposing vesicular GABA transporter-immunoreactive (vGAT-ir) puncta with GnRH neurons within the rPOA was counted in VEH control male (n = 5) and PNAM mice (n = 5). GnRH-GFP transgenic^37^ mice enabled us to count the number of putative vGAT contacts to the GnRH cell soma and first 75 µm of the primary dendrite (Fig. 5A; i,ii & 5B; i,ii). Omission of the rabbit anti-vGAT antibody resulted in the absence of vGAT-ir puncta in our negative control (Supplementary Fig. S1).

There was no significant difference in the density of closely apposed vGAT-ir puncta to the GnRH neuronal soma between VEH control (n = 5) and PNAM (n = 5) mice (Fig. 5A–C; *p* = 0.82). Additionally, the density of closely apposed vGAT-ir puncta with the primary dendrite of GnRH neurons was not significantly different (Fig. 5A–C, *p* = 0.50). In addition to calculating the density of contacts, the location of vGAT  contacts  to GnRH neurons was also determined. Similar to density, the total number of counted vGAT appositions to the GnRH neuron soma was not significantly different between VEH control and PNAM mice (Fig. 5D, Supplementary Table. S1). Likewise, the number of vGAT appositions to any region of the proximal dendrite was not significantly different between VEH control and PNAM mice (Fig. 5D, Supplementary Table S1). As a positive control, vGAT appositions to GnRH neurons were also investigated in the female littermates of VEH control and PNAM mice. As expected, the density of closely apposed vGAT-ir puncta with the GnRH neuronal soma and primary dendrite was significantly increased in PNA females (n = 4) compared to VEH control (n = 4) females (Supplementary Fig. S3, Table S3).

## Discussion

**Figure 6:**
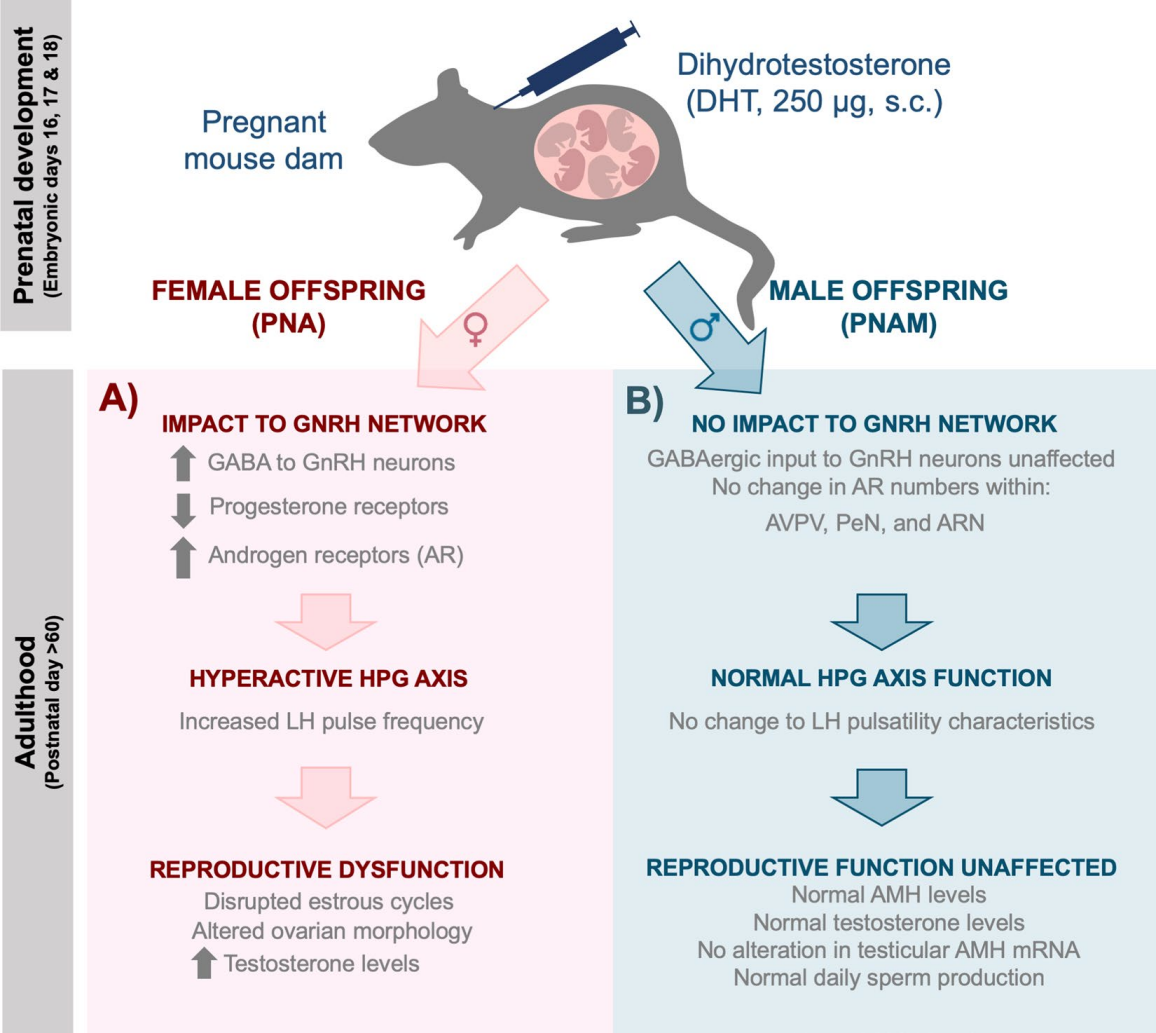
Summary of the effect of maternal androgen excess on the adult reproductive function of male and female offspring. DHT administration in late gestation (embryonic days 16, 17 and 18) has differential effects on the adult reproductive function of female (**A**) and male (**B**) offspring. Female offspring exposed to elevated maternal androgens manifest an altered GnRH neuronal network (i.e. enhanced GABAergic input to GnRH neurons and increased hypothalamic AR expression), a hyperactive HPG axis (i.e. increased LH pulse frequency) and reproductive dysfunction (i.e. disrupted estrous cycles and elevated testosterone levels)^20,22–24^. In contrast, male offspring also exposed to elevated maternal androgens do not manifest any alteration in their GnRH neuronal network (i.e. specifically GABAergic input to GnRH neurons and AR expression throughout various hypothalamic and limbic regions), exhibit normal HPG axis function and ultimately normal reproductive function (i.e. normal daily sperm production and other parameters of testicular function).

We found that, in stark contrast to females, males exposed to prenatal androgen excess do not exhibit any identifiable impairments in adult reproductive function (Fig. 6). Testicular function and gonadal hormone secretion in PNAM mice were not different to controls. Unlike their female littermates^23^, PNAM mice did not exhibit any alterations in LH pulsatility, suggesting no significant alterations in HPG axis function. In support of this, hypothalamic expression of AR and the density of vGAT contacts with GnRH neurons was not affected by elevated maternal DHT levels. Collectively, our findings demonstrate that elevated non-aromatisable androgen exposure late in gestational development has no discernible effect on the reproductive axes of male mice.

The maternal androgen excess protocol used in the current study involved delivering DHT in late gestation (ED 16–18). The use of a non-aromatisable androgen largely isolates androgen receptor-mediated effects and avoids the confounds of estrogenic effects by testosterone metabolites. This protocol has been shown to elicit a lean PCOS-like phenotype of reproductive dysfunction in female offspring by multiple groups^20,22–24,38–41^. However, these data demonstrate that the developing male reproductive axis remains protected from this excess androgen signalling. Our findings are similar to those of Dean *et al*.^42^ who found that male reproductive tract development in the rat was unaffected by prenatal DHT exposure during this masculinisation programming window. The reported programming effect of PNA on the adult metabolic and reproductive health of male primates^27^ and rams^33^ could be explained by the administration of aromatisable testosterone and downstream effects of metabolites like estradiol, as well as the timing of exposure.

In contrast to our findings here, prenatally androgenised rams exhibit increased basal serum LH levels and LH responsiveness to GnRH^33^. As noted above, this difference is likely due to treatment paradigm. Prenatal androgen exposure of the developing ram was targeted to early and mid-gestational development, while prenatal androgen exposure here was delivered in late gestation. Although both studies targeted the period in which brain sexual differentiation is established^43^, androgen exposure was prolonged in rams and delivered over a relatively short duration in mice. However, differences in these studies are more likely due to the delivery of testosterone propionate compared to DHT. Together, these findings suggest that excessive estrogenic testosterone metabolites elicit programming responses in the developing male HPG axis that are not apparently driven by androgen receptor mediated actions alone.

However, we cannot discount technical limitations of our approach to measure LH. Measuring pulsatile LH secretion in males, and rodents in particular, is challenging due to the slow frequency of pulsatile secretion^44–47^. Although serial blood sampling for LH was performed in all PNAM (n = 10) and VEH control mice (n = 10), only five VEH control and four PNAM mice exhibited at least one LH pulse within the four-hour experimental period. Therefore, due to the physiological constraints of male LH pulse frequency, the number of animals per group analysed in this portion of work was low. Although no clear alterations in LH pulse frequency were identified, the suggestion of elevated LH AUC could indicate subtle changes in pituitary responsiveness. It could be of interest in future studies to investigate this responsiveness and determine if there are any changes in GnRH receptor or LH-beta subunit expression on gonadotropes.

Supporting the lack of change in gonadotropin signalling^48^, testosterone levels in PNAM mice were not different to controls. In contrast, Recabarren *et al*.^41^ found that prenatally androgenised rams exhibit elevated basal plasma testosterone concentrations. Again, this discrepancy is likely due to differences in the timing, duration and/or level of androgen exposure. Our data suggests that males exposed to elevated non-aromatisable androgens for a relatively short duration in late prenatal development do not manifest altered adult Leydig cell function. Of interest, sons of women with PCOS examined over pubertal development exhibit higher cholesterol and LDL levels, but do not present with any differences in circulating concentrations of LH, FSH sex hormone-binding globulin, testosterone, androstenedione (A4), 17α-hydroxyprogesterone (17-OHP) or AMH^49^.

Unlike prepubertal sons and adult male relatives of PCOS women that demonstrate increased plasma AMH levels^25,26^, we found no difference in plasma AMH levels between PNAM and VEH control mice in adulthood. In addition, PNAM mice did not exhibit any discernible alterations in testicular mRNA levels of *Amh* or its receptor variants. As increased AMH levels reflect increased Sertoli cell number or function, these data suggest that maternal androgen excess of the magnitude used in the present study is not sufficient to impact AMH secretion or alter Sertoli cell function. Thus, the reported elevation in AMH levels in male PCOS relatives likely reflects more pronounced androgen exposure or is genetically heritable^26,50^.

Adult testicular gametogenesis, determined by daily sperm production (DSP)^51^, also remained unaffected. This is strikingly different to the impact of prenatal DHT excess on the adult female mouse ovary^22–24^. This is also in contrast to the effect of elevated maternal testosterone in the ram. Rams born to ewes injected with testosterone propionate exhibit significantly reduced sperm count and mean straight line motility from 26–40 weeks of age^28^. Additionally, post-pubertal rams (~7 months) born to ewes injected with testosterone propionate during early gestational development (GD 30–58) manifest significantly decreased sperm concentration^30^. However, these effects are again likely to be associated with the activity of estrogenic testosterone metabolites, as “extra” prenatal DHT exposure in the rat showed a similar lack of impact on male reproductive tract development^42^.

Androgens exert their effects via the androgen receptor (AR), a ligand dependent nuclear transcription factor^52^ that is highly regulated by circulating androgen levels^53,54^. Testosterone propionate administration to gonadectomised mice results in a significant linear trend in AR expression up regulation within various regions of both male and female brains^53^. PNA-treated female mice are hyperandrogenic and exhibit increased AR-positive cells within the AVPV, an important hypothalamic region for regulating female ovulatory cycles in rodent species^23^. In line with the lack of changes observed in plasma testosterone levels in PNAM mice, the number of AR expressing cells was not different in the AVPV, PeN or any region of the ARN, hypothalamic regions of known GnRH neuron afferents^36^.

Despite the maintenance of reproductive health in PNAM mice, is was of interest to determine whether maternal DHT impacted the GABAergic wiring to the GnRH neurons in a similar manner to that identified in females^23,24^. To address this, the density of immunoreactive vGAT puncta^55^ in contact with GFP-expressing GnRH neurons was assessed with high resolution confocal imaging to identify presynaptic GABA terminals associated with GnRH neurons^23,24,56^. It has been reported previously that approximately 80% of VGAT puncta identified in close association with GnRH neurons are co-expressed with the presynaptic marker synaptophysin^56^. Here, while PNA female littermates serving as positive controls demonstrated the expected increase in putative GABAergic input to GnRH neuron, this anatomical circuitry was unaffected in PNAM mice. This aligns with findings showing that spontaneous GABAergic transmission frequency to GnRH neurons is also unchanged in PNAM mice^38^. Although, of interest, PNAM mice do exhibit increased GABAergic transmission to GnRH neurons at 3 weeks of age, just prior to the onset of puberty^38^, a time point associated with increased GABAergic innervation and transmission in PNA females^24,38^. Unmodified GABAergic inputs to GnRH neurons in adult PNAM mice is consistent with the absence of evidence for any impairments in the male reproductive axis.

These results are particularly interesting as they further support the idea that enhanced GABAergic wiring in PNA females is not a mere “masculinisation” of the associative GnRH neuronal circuitry^23,24,56^. Elevated DHT during late prenatal development programmes changes in GnRH neuronal network of females, while males remain protected from the development of abnormal wiring. These findings highlight the exquisite sensitivity of the developing female brain, which ordinarily is exposed to very low androgens during development, and suggest that the male brain, which in late gestation is exposed to elevated gonadal testosterone, is able to compensate. The male reproductive axis is functional before birth in male foetuses, and maintains homeostatic levels of serum testosterone via negative feedback mechanisms^12,57,58^. Therefore, it is plausible that in males the active hypothalamic-pituitary-testicular axis is able to compensate for exogenous androgen exposure during prenatal development by adjusting LH secretion from the anterior pituitary and downstream testosterone synthesis to consequently restore homeostasis to the androgen milieu *in utero*. As such, in the present study, DHT administration during this period of prenatal development is able to elevate androgen levels in female foetuses to pathological levels, but not in male foetuses as they are capable of hormonal compensation.

In conclusion, we provide here a collective body of reproductive and neuroendocrine evidence illustrating that exposure to elevated maternal non-aromatisable androgens during late gestational development does not elicit any notable impairment in male reproductive function in mice. We have shown that PNA treatment in males has no detrimental influence on adult reproductive function, unlike that seen in their female counterparts. It remains possible that exposure to elevated maternal androgens for a longer time-frame or earlier in gestational development may programme long lasting abnormalities, accounting for the observed reproductive hormonal abnormalities reported in the male relatives of PCOS women.

## Materials and Methods

### Animal ethics

All protocols and procedures were approved by the University of Otago Animal Ethics committee (Dunedin, New Zealand) and all experiments were performed in accordance with relevant guidelines and regulations.

### Mice

Homozygous GnRH-GFP mice^37^ of a C57BL/B6 background were housed with *ad libitum* access to food and water, and maintained under a 12-hour light/dark cycle. To generate prenatally androgenised male mice (PNAM), we used established protocols as previously described^22–24^. Briefly, pregnant mouse dams were subcutaneously injected with 100 μL of DHT (250 µg) in sesame oil to generate PNAM mice, or sesame oil alone (to generate VEH control male offspring) on gestational days 16, 17 and 18. VEH control (n = 10) and PNAM (n = 10) mice were then analysed in adulthood (postnatal day > 60). Groups were comprised of the male offspring from 3 VEH-treated litters and 3 DHT-treated litters. VEH female littermates displayed normal estrous cyclicity and DHT female littermates were acyclic confirming the expected female PNA phenotype^23,24^ and treatment effect in all litters.

### Pulsatile LH measurements

VEH control (n = 10) and PNAM (n = 10) mice were habituated with daily handling for 3 weeks. As previously reported^59^, 4 μL of blood was collected from the tail tip in 10 minute intervals for 4 hours (between 9:00 and 1:00 pm), diluted in PBS-Tween and then immediately frozen. LH levels were determined, as previously described^23^, using the mouse LH-RP reference provided by Albert F. Parlow (National Hormone and Pituitary Program, Torrance, California, USA)^23,24^. The ELISA assay sensitivity was 0.04 ng/mL, the intra-assay coefficient of variation was 6% and the inter-assay coefficient of variation of the ELISA was 14%. LH pulses were characterised by (1) LH pulse values above 255% of the preceding value^35^, (2) fitting a typical LH pulse shape (i.e. immediate rise in LH value with at least two succeeding decaying points down)^60^ and (3) confirmed with the software DynPeak^61^.

### Gonadal hormone measurements

Commercially available ELISA kits were used to measure plasma levels of testosterone (LDN GmbH&Co.KG, Nordhorn, Germany, cat# AR E-8000) and AMH (Ansh Labs, Texas, USA, cat# AL-113) according to the manufactures’ instructions as used previously^23,24^. The sensitivity of the testosterone ELISA assay was 0.66 ng/mL at the two standard deviation confidence limit and the intra-assay coefficient of variation was 7%. The sensitivity of the AMH ELISA assay was 0.023 ng/mL and the intra-assay coefficient of variation was 3.4%.

### Daily sperm production (DSP) analysis

To measure DSP, we adapted and optimised a protocol previously used by others^51^. Following removal of the tunica albuginea, the fresh testis segment used for DSP analysis was weighed. To this segment, 10 μL of saline-merthiolate-triton (SMT) buffer (0.9% NaCl, 0.05% Trition-X-100) was added per mg of testicular tissue followed by homogenisation with a Dounce homogeniser for 3 minutes. The homogenate (100 µL) was then diluted in 500 μL of SMT buffer and 10 μL of this final solution was loaded onto a haemocytometer grid. Five minutes after loading the final homogenate solution (allowing for the spermatozoa heads to settle at the bottom of the haemocytometer), the number of spermatozoa heads visible under a Zeiss epifluorescent Axioplan microscope at 20× magnification (Zeiss Corporation, Oberkochen, Germany) was counted on two haemocytometer quadrants. An averaged spermatozoa value was calculated from the two separate spermatozoa counts from quadrant 1 and 2. Following a series of calculations to calculate the number of spermatozoa in both testes (Supplementary Fig. 2), this value was divided by 4.84 (the number of days developing spermatids spend in steps 14–16 of the mouse sperm maturation process) to provide an overall DSP value.

### Testicular mRNA reverse-transcriptase qPCR

Testis segments were frozen and stored at −80 °C before homogenisation in TRI reagent (Sigma-Aldrich, catalogue T9424) to isolate RNA. mRNA (1 µg) was treated with 2 units of DNase (TurboDNase, ThermoFisher Scientific, cat# AM2238) for 30 minutes at 37 °C and then followed by inactivation for 5 minutes at 75 °C. The Revertaid reverse-transcription kit (ThermoFisher Scientific, Massachusetts, USA, cat# K1622) was utilised to convert RNA to cDNA. The qPCR was conducted with a LightCycler480 (Roche, Switzerland) cycling first through a preincubation heating period of 50 °C for 20 seconds and 95 °C for 15 seconds. Then an amplification period was completed, with 50 cycles of 95 °C for 20 seconds, 60 °C for 20 seconds and 73 °C for 30 seconds. Melt curve acquisition was then performed with a single cycle of 95 °C for 5 seconds and 65 °C for 1 minute. Finally, a cooling cycle was conducted of 40 °C for 30 seconds. Following the completion of all cycles, data acquisition was performed obtaining the crossing point-PCR cycle (CP) values for all samples, melting curves, amplification curves, and derivatised melt curves. Relative mRNA expression of each transcript of interest (*Amh*, *Amh* receptors; *Amhr2*, *Acvr1*, *Bmpr1a*, *Bmpr1b*) was calculated according to the formula 2^−ΔCp^ with β-actin (*Actb*) used as the reference gene. Primer sequences are provided in Supplementary Table 3.

### Brain collection and sectioning

At postnatal day 72–95, all VEH control and PNAM mice underwent a transcardial perfusion of 4% paraformaldehyde (PFA). The resulting perfusion-fixed brains were dissected from the skull, post-fixed in 4% PFA overnight and then cyroprotected in a 30% sucrose/Tris-Buffered saline (TBS) solution for a further 24 hours at 4 °C. The perfusion-fixed brains were then cut into 30 μm thick sections using a freezing microtome.

### Chromagen immunohistochemistry

Coronal brain sections of the AVPV, PeN, rARN, mARN and cARN were selected with reference to the Paxinos mouse brain atlas^62^. Free-floating immunohistochemistry was performed as previously reported^23,24,63^, with primary antibody omission serving as the negative control. The primary antibody polyclonal rabbit anti-AR antibody (AR, PG-21, Millipore, catalogue 06–680) was used at a concentration of 1: 2,500. The secondary antibody biotinylated goat anti-rabbit (Vector Laboratories Inc., catalogue BA-1000) was used at a concentration of 1:800. Chromagen labelling was achieved using the A/B Vectastain Elite kit (1:200, Vector Laboratories Inc., CA, USA, cat#PK-4000) and 3,3’-diaminobenzidine (DAB) enhanced with nickel.

### Fluorescent immunohistochemistry

Coronal brain sections of the rPOA (including the organum vasculosum of lamina terminalis, OVLT) were selected with reference to the Paxinos mouse brain atlas^62^. The primary antibodies of polyclonal rabbit anti-vGAT (Synaptic Systems, catalogue 131003) and polyclonal chicken anti-GFP (Aves Lab Inc., catalogue GFP-1020) were used at the respective concentrations of 1:750 and 1: 5,000. The following secondary antibodies of Rhodamine Red^TM^ donkey anti-rabbit 568 (Jackson ImmunoResearch Laboratories Inc., catalogue 711295152) and Alexa Fluor donkey anti-chicken 488 (Jackson ImmunoResearch Laboratories Inc., catalogue 703545155) were used at the respective concentrations of 1: 1,000 and 1:500.

### Microscopy and image analysis

Microscopy image acquisition was performed using an Olympus BX-51 microscope (Olympus corporation, Tokyo, Japan) or multi-photon Nikon AR1 confocal microscope. Chromagen labelling of AR receptors was imaged with brightfield light microscopy using a 10× objective, and ImageJ software (National Institutes of Health, Bethesda, Maryland, USA) was used to assist in the quantification of the number of AR-positive nuclei in two representative sections from each nucleus analysed (AVPV, PeN, rARN, mARN and cARN).

Following double immunofluorescent labelling of vGAT and GFP in GnRH-GFP VEH control and PNAM mice, 10 GnRH neurons were selected at random from two representative sections of the rPOA. GnRH neurons were imaged using a Nikon A1R multi-photon confocal microscope with 488 (HV: 80, Offset: 0, Laser: 1.5) and 543 nm (HV: 85, Offset: −5, Laser: 2.0) diode lasers. Sections were imaged using a Plan NeoFluor 40× oil objective, taking Z-stack images every 0.6 µm (pinhole at 1.1 AU) with a 2× zoom function (Nikon® Instruments Inc., Tokyo, Japan). As previously described, vGAT apposition and GnRH spine density was analysed at the GnRH neuron soma and in 15 μm intervals of the primary dendrite out to 75 μm^23,24^.

### Statistical analysis

All statistical analysis was performed with the statistical software R® (R Core Team, Vienna, Austria). Statistical significance was determined as *p* < 0.05. Statistical analysis for normality of distribution (conducted with a Shapiro-Wilk normality test) and homogeneity of variance (conducted with a Fisher’s test) was initially conducted to ensure the appropriate selection of statistical tests. If both normality of distribution and homogeneity of variance was verified, then a Student’s t-test was conducted. If either normality of distribution or homogeneity of variance (or both) was not verified, then a Wilcoxon rank sum test was conducted. All data are presented as mean ± standard error of the mean (SEM) in graphs generated with GraphPad Prism v7.02® software (Graphpad Software Inc., La Jolla, CA, USA).

## Supplementary information

Supplementary Information

## Data Availability

The datasets generated during and/or analysed during the current study are available from the corresponding author on reasonable request.
