# Supplementary material for: The influence of maternal androgen excess on the male reproductive axis
Source: Sci Rep. 2019 Dec 11;9:18908. doi: 10.1038/s41598-019-55436-9 (PMC6906411; doi:10.1038/s41598-019-55436-9)
Supplement: Supplementary file 1 — Supplementary Information [file 41598_2019_55436_MOESM1_ESM.docx]

**The influence of maternal androgen excess on the male reproductive axis**

**Authors:** Sarah Holland^1^, Melanie Prescott^1^, Michael Pankhurst^2^ and Rebecca E. Campbell^1^

^1^Centre for Neuroendocrinology and Department of Physiology, School of Biomedical Sciences, University of Otago, Dunedin, New Zealand, 9054

^2^Department of Anatomy, School of Biomedical Sciences, University of Otago, Dunedin, New Zealand, 9054

**Supporting Information**

**Table S1.** Raw number of counted vGAT appositions in VEH control and PNAM mice.

| **Regions of GnRH neuron** | **VEH control male (n = 5)** | **PNAM (n = 5)** | ***p* value** |
| --- | --- | --- | --- |
| **Soma** | 4.15 ± 0.22 | 4.26 ± 0.12 | 0.70; Student’s t-test. |
| ***Segments of the GnRH primary dendrite*** | | | |
| **0 – 15 µm** | 4.04 ± 0.29 | 4.32 ± 0.29 | 0.46; Wilcoxon rank sum test. |
| **15 – 30 µm** | 3.40 ± 0.38 | 3.57 ± 0.31 | 0.74; Student’s t-test. |
| **30 – 45 µm** | 2.4 ± 0.32 | 3.04 ± 0.30 | 0.18; Student’s t-test. |
| **45 – 60 µm** | 3.09 ± 0.24 | 3.53 ± 0.25 | 0.24; Student’s t-test. |
| **60 – 75 µm** | 3.01 ± 0.27 | 3.37 ± 0.19 | 0.24; Student’s t-test. |

**Table S2.** Raw number of counted vGAT appositions in VEH control and PNAM mice.

| **Regions of GnRH neuron** | **VEH control female (n = 4)** | **PNA female (n = 4)** | ***p* value** |
| --- | --- | --- | --- |
| **Soma** | 5.72 ± 0.51 | 7.62 ± 0.27 | 0.03; Student’s t-test |
| ***Segments of the GnRH primary dendrite*** | | | |
| **0 – 15 µm** | 5.3 ± 0.61 | 7.62 ± 0.58 | 0.03; Student’s t-test |
| **15 – 30 µm** | 4.15 ± 0.40 | 6.53 ± 0.36 | 0.004; Student’s t-test |
| **30 – 45 µm** | 4.73 ± 0.52 | 6.29 ± 0.17 | 0.03; Student’s t-test |
| **45 – 60 µm** | 3.44 ± 0.33 | 4.09 ± 0.40 | 0.76; Student’s t-test |
| **60 – 75 µm** | 3.62 ± 0.40 | 3.94 ± 0.39 | 0.59; Student’s t-test |

**Table S3.** Primer sequences for qPCR of testicular mRNA transcripts in VEH control (n = 10) and PNAM mice (n = 10).

| **Gene** | **Abbreviation** | **Accession Number** | **PCR product length**  ***(bp = base pairs)*** | **Primer Sequence** | | **References** |
| --- | --- | --- | --- | --- | --- | --- |
| Anti-Müllerian hormone | *Amh* | NM_007445.2 | 118bp | Forward | 5’TGCGCGAGCTGAGTGTAGATCT3’ | ^1,2^ |
|  |  |  |  | Reverse | 5’TAGCGCGGATTACGGTCAGACT3’ |  |
| AMH Receptors | | | | | | |
| Anti-Müllerian hormone receptor 2 | *Amhr2* | NM_144547.2 | 89bp | Forward | 5’GCTCCAGAGCTCTTGGACAA3’ | ^2,3^ |
|  |  |  |  | Reverse | 5’AGTAGTAGCGCCAGAGAGTAAA3’ |  |
| Activin A receptor type 1 | *Acvr1* | NM_001110204.1 | 274bp | Forward | 5’GATCAACAGAGGCCAAACATACCTA3’ | ^2,4^ |
|  |  |  |  | Reverse | 5’AGATGGATTCTGTTCTGACAACCA3’ |  |
| Bone morphogenetic protein receptor type 1A | *Bmpr1a* | NM_009758.4 | 159bp | Forward | 5’GTCTATTCCAGGGCAGATTTCCTA3’ | ^2,5^ |
|  |  |  |  | Reverse | 5’CCTGCTTAACATCTGACGCAAGT3’ |  |
| Bone morphogenetic protein receptor type 1B | *Bmpr1b* | NM_001277216.1 | 277bp | Forward | 5’ATACCAGCTTCCCTATCACGACCT3’ | ^2,6^ |
|  |  |  |  | Reverse | 5’TGAAATTCTTGCTCTGTCCACAAGTA3’ |  |
| Reference gene | | | | | | |
| Beta-actin | *Actb* | NM_007393.5 | 260bp | Forward | 5’GGTACCACCATGTACCCAGG3’ | ^2,7^ |
|  |  |  |  | Reverse | 5’GAAAGGGTGTAAAACGCAGC3’ |  |

| **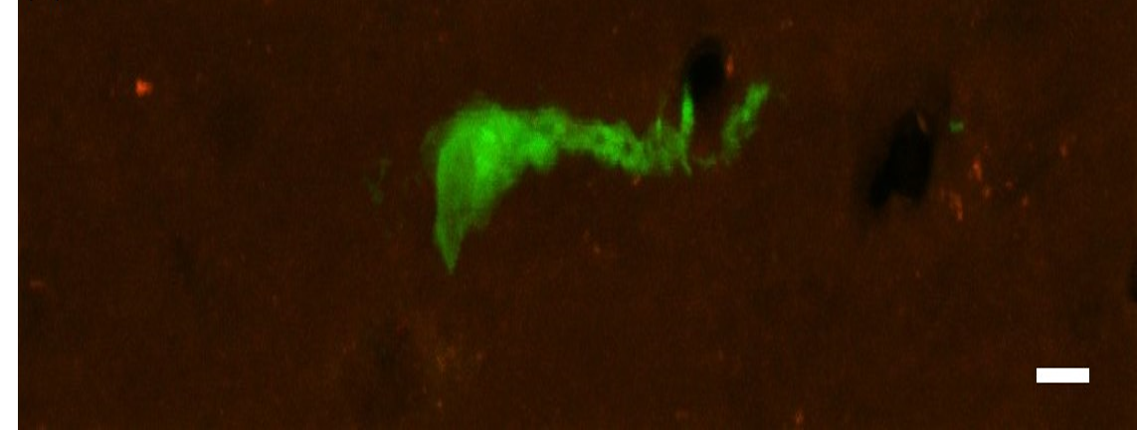** |
| --- |
| **Figure S1.** Omission of vGAT and GFP antibody as a negative control. Projected Z-stacked confocal image of a representative negative control section showing endogenous GFP expression in a GnRH neuron and the absence of vGAT-ir puncta (16.8 µm optical thickness). Scale bars = 5 µm. |

| **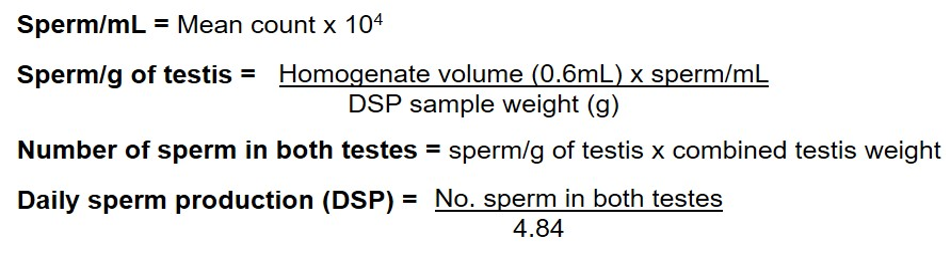** |
| --- |
| **Figure S2.** Daily Sperm Production (DSP) calculations. The series of calculations that was performed to obtain a DSP value for each individual male mouse. |

| **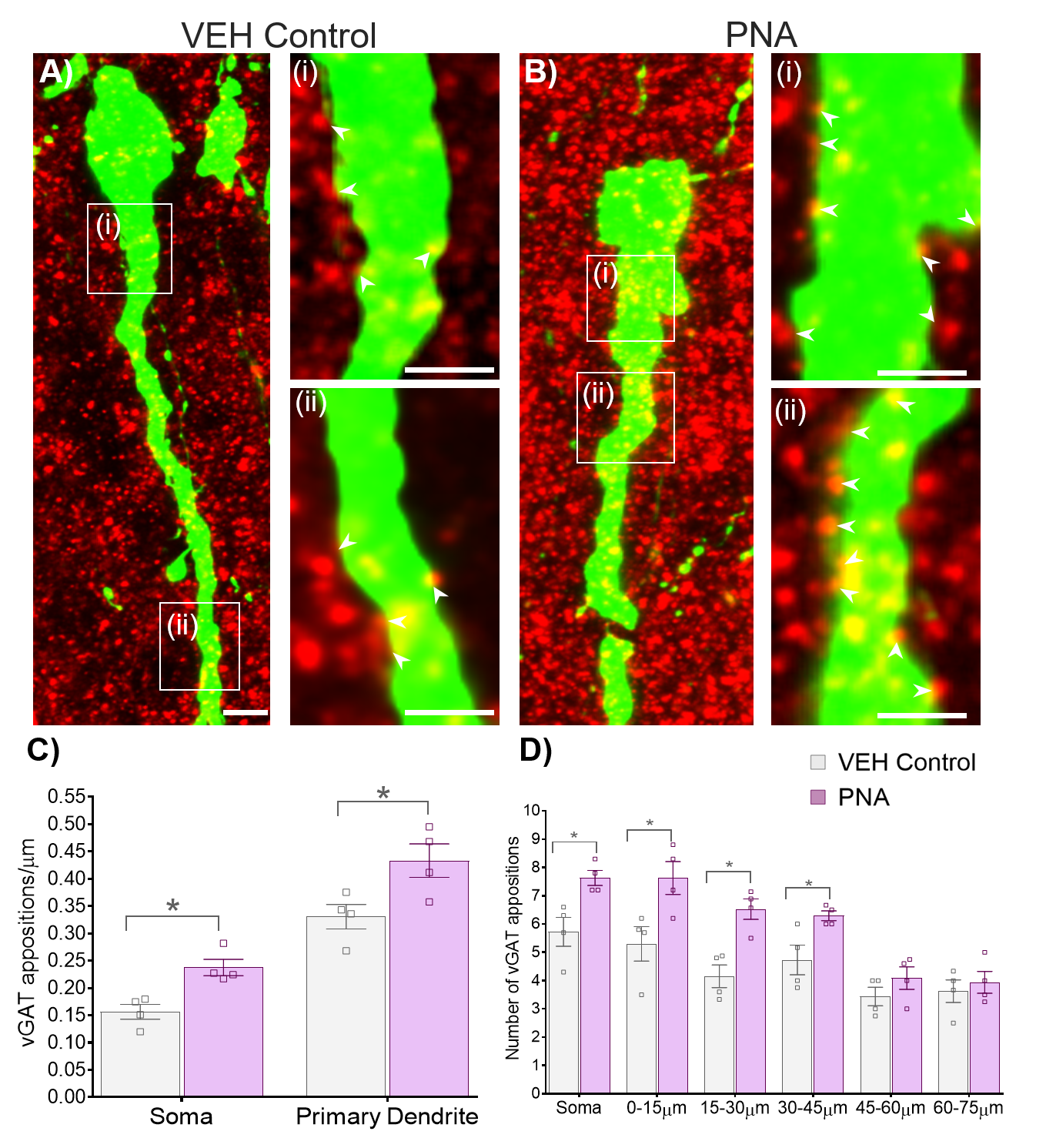** |
| --- |
| **Figure S3.** GABAergic apposition density to GnRH neurons and the number of counted vGAT appositions to GnRH soma and first 45 µm of the primary dendrite is significantly increased in PNA females. The density of vGaT appositions at the soma (**C**) was significantly increased in PNA (n = 4) animals compared to VEH control females (n = 4) (Student’s t-test, *p* value = 0.013). Likewise, the density of vGAT appositions at the primary dendrite (**C**) was significantly increased in PNA animals compared to VEH control females (Student’s t-test, *p* value = 0.035). The number of vGaT appositions to GnRH neurons in PNA mice was significantly increased at the GnRH cell soma (Student’s t-test, *p* value = 0.03) and to the 0-15 µm segment (Student’s t-test, *p* value = 0.03), 15-30 µm segment (Student’s t-test, *p* value = 0.004) and 30-45 µm segment (Student’s t-test, *p* value = 0.03) of the primary dendrite (**B**). Values are presented as mean ± SEM. * *p* < 0.05. |

**References**

1 Behringer, R. R., Cate, R. L., Froelick, G. J., Palmiter, R. D. & Brinster, R. L. Abnormal sexual development in transgenic mice chronically expressing mullerian inhibiting substance. *Nature* **345**, 167-170, doi:10.1038/345167a0 (1990).

2 Pankhurst, M. W. *et al.* Anti-Mullerian hormone overexpression restricts preantral ovarian follicle survival. *The Journal of endocrinology* **237**, 153-163, doi:10.1530/joe-18-0005 (2018).

3 Mishina, Y. *et al.* Sequence, genomic organization, and chromosomal location of the mouse Mullerian-inhibiting substance type II receptor gene. *Biochemical and biophysical research communications* **237**, 741-746, doi:10.1006/bbrc.1997.7224 (1997).

4 Schmitt, J., Mielke, R. & Schrewe, H. Genomic organization of a mouse type I activin receptor. *Biochemical and biophysical research communications* **213**, 211-217, doi:10.1006/bbrc.1995.2118 (1995).

5 Mishina, Y. *et al.* Genomic organization and chromosomal location of the mouse type I BMP-2/4 receptor. *Biochemical and biophysical research communications* **206**, 310-317, doi:10.1006/bbrc.1995.1043 (1995).

6 ten Dijke, P. *et al.* Characterization of type I receptors for transforming growth factor-beta and activin. *Science* **264**, 101-104 (1994).

7 Barratt, K. S., Diamand, K. E. M. & Arkell, R. M. Identification of reference genes suitable for RT-qPCR studies of murine gastrulation and patterning. *Mammalian genome : official journal of the International Mammalian Genome Society* **29**, 656-662, doi:10.1007/s00335-018-9769-0 (2018).
